# Brain endothelium-derived extracellular vesicles containing amyloid-beta induce mitochondrial alterations in neural progenitor cells

**DOI:** 10.20517/evcna.2022.22

**Published:** 2022-12-01

**Authors:** Olivia M. Osborne, Jennifer M. Kowalczyk, Kelssey D. Pierre Louis, Manav T. Daftari, Brett M. Colbert, Oandy Naranjo, Silvia Torices, Ibolya E. András, Derek M. Dykxhoorn, Michal Toborek

**Affiliations:** ^1^Department of Biochemistry and Molecular Biology, University of Miami Miller School of Medicine, Miami, FL 33136, USA.; ^2^Dr. JT Macdonald Foundation Biomedical Nanotechnology Institute of the University of Miami, University of Miami Miller School of Medicine, Miami, FL 33136, USA.; ^3^Medical Scientist Training Program, University of Miami Miller School of Medicine, Miami, FL 33136, USA.; ^4^Dr. John T. Macdonald Foundation Department of Human Genetics, John P. Hussman Institute for Human Genomics, University of Miami Miller School of Medicine, Miami, FL 33136, USA.

**Keywords:** Blood-brain barrier, mitochondrial bioenergetics, extracellular vesicle, neurogenesis, Alzheimer’s disease, Seahorse, neural progenitor cell

## Abstract

**Aim:**

Elevated brain deposits of amyloid beta (Aβ_40_) contribute to neuropathology and cognitive dysfunction in Alzheimer’s disease (AD). However, the role of the blood-brain barrier (BBB) as an interface for the transfer of Aβ_40_ from the periphery into the brain is not well characterized. In addition, a substantial population of neural progenitor cells (NPCs) resides in close proximity to brain capillaries that form the BBB. The aim of this study is to understand the impact of brain endothelium-derived extracellular vesicles (EV) containing Aβ_40_ on metabolic functions and differentiation of NPCs.

**Methods:**

Endothelial EVs were derived from an *in vitro *model of the brain endothelium treated with 100 nM Aβ_40_ or PBS. We then analyzed the impact of these EVs on mitochondrial morphology and bioenergetic disruption of NPCs. In addition, NPCs were differentiated and neurite development upon exposure to EVs was assessed using the IncuCyte Zoom live cell imaging system.

**Results:**

We demonstrate that physiological concentrations of Aβ_40_ can be transferred to accumulate in NPCs via endothelial EVs. This transfer results in mitochondrial dysfunction, disrupting crista morphology, metabolic rates, fusion and fission dynamics of NPCs, as well as their neurite development.

**Conclusion:**

Intercellular transfer of Aβ_40_ is carried out by brain endothelium-derived EVs, which can affect NPC differentiation and induce mitochondrial dysfunction, leading to aberrant neurogenesis. This has pathological implications because NPCs growing into neurons are incorporated into cerebral structures involved in learning and memory, two common phenotypes affected in AD and related dementias.

## INTRODUCTION

Alzheimer’s disease (AD) is the most common neurodegenerative disease, affecting one in every eight people over the age of 65 and around half of the individuals over the age of 85^[1]^. Elevated brain deposits of beta-amyloid (Aβ) are now accepted to contribute to the neuropathology and cognitive dysfunction seen in AD^[2]^. The levels of Aβ in the central nervous system (CNS) result from an equilibrium between production in the brain, influx from plasma, and efflux via blood-brain barrier (BBB) efflux transporters^[3]^. Although these processes contribute to the formation of Aβ deposits and plaques in AD, emerging evidence indicates that the BBB is a critical interface in the transfer of Aβ from the periphery into the brain^[4,5]^. However, the mechanisms underlying this process remain unclear. Recent evidence indicates that it is important to consider the role of the BBB in the transfer of Aβ when analyzing neuropathologies associated with AD^[6-9]^. We previously demonstrated that exposure to EVs carrying Aβ_40_ (EV-Aβ_40_) negatively affects the differentiation of NPCs into mature neurons; however, the mechanisms behind these effects are poorly understood^[10,11]^. In the present study, we hypothesize that an underlying cause of altered neurogenesis, which is a prominent feature of AD, may be mitochondrial dysfunction that results from EV-mediated Aβ_40_ exposure. The effect of exposing NPCs to Aβ_40_ that may be trafficked across the BBB, compounded with endogenous production of Aβ_40_ in the brain, may exacerbate the mitochondrial dysfunction observed in progenitor cells, leading to aberrant neurogenesis.

The BBB provides a physiological, immunological, and structural interface between the brain parenchyma and the peripheral circulation. It also represents a crucial interface between the circulatory system and brain tissue, helping to maintain the overall homeostasis of the CNS. The brain endothelium, formed by cerebral endothelial cells, is the crucial element of the BBB that is responsible for its structural and metabolic properties. These endothelial cells produce EVs that can travel and interact with other cell types in the brain to deliver various biologically active cargo materials, such as Aβ_40_^[10,11]^. Aβ_40_ is produced by the cleavage of amyloid precursor protein (APP), which is ubiquitously expressed on the membrane of neurons as well as endothelial cells that make up the BBB^[12,13]^. CNS levels of Aβ_40_ result from an equilibrium established between production in the brain, influx from plasma, and efflux by BBB transporters. We here study the involvement of Aβ_40_ because it is the main isoform produced by the cleavage of APP, making up 90% of all Aβ_40_ produced in the brain. Increased Aβ_40_ deposition is also associated with vascular dysfunction and is likely to compromise the integrity of the BBB during AD^[14,15]^. Therefore, our studies focus on this amyloidogenic species along with its ability to cross the BBB and impair neurogenesis via EV-mediated transfer. 

EVs have recently been postulated to be significantly involved in various neurodegenerative diseases, including Aβ_40_ pathology^[16,17]^. It was demonstrated that Aβ_40_ could be packaged into EVs and shed into the microenvironment and that human amyloid plaques are enriched with EV proteins^[9]^. Additionally, we previously showed that exposure of brain endothelial cells to Aβ_40_ results in effective packaging of Aβ_40_ in EVs, leading to the transfer of Aβ_40_ into neighboring cells, including NPCs. EVs are heterogeneous in their shape, size, origin, and content. They include exosomes with a size of approximately 30-100 nm and other larger vesicles^[18,19]^. EVs are formed in a stepwise process whereby the vesicles bud from the endosomal membranes generating intra-endosomal vesicles, followed by fusion of the endosome membrane with the plasma membrane and release of the EVs into the extracellular environment. Their content is diverse and includes mRNAs, miRNAs, lipids, and proteins^[20]^. EV cargo can be released into the immediate microenvironment or peripheral sites when transported via the circulatory system^[21]^. EVs, being of endosomal origin, have been shown to be released from multiple cell types creating a unique cell-to-cell communication system, which may play important physiological and pathological roles in the CNS^[22,23]^. However, few studies have focused on EVs derived from brain endothelial cells, or on the effect of their cargo on neurogenesis in the brain. This process is of physiological significance because neurogenic niches are predominantly localized around the brain microvasculature that forms the BBB^[24]^. Although Aβ_40_ accumulation has a detrimental effect on neuronal growth in diseases such as AD, the mechanism of action that leads to aberrant neurogenesis remains largely unknown. Therefore, the objective of the present study is twofold: (1) to determine the mechanistic effect of Aβ_40_ on NPCs that may lead to aberrant neurogenesis; and (2) to delineate whether vesicle-mediated trafficking of Aβ_40_ can potentiate the neurogenic deficiency seen in AD patients. Our results demonstrate that brain endothelium-derived EVs can transfer cargo to NPCs and may serve as a vehicle for cell-to-cell communication that regulates mitochondrial metabolic dynamics in NPCs and, ultimately, decreases their neurogenic potential.

## METHODS

### Cell cultures

#### hCMEC/D3

The human cerebral microvascular endothelial cells (hCMEC/D3) used in this study represent a robust, well-characterized, and stable human brain endothelial cell line. hCMEC/D3 cells were cultured on rat tail collagen type I (Thermo Fisher Scientific, Cat# CB-40236)-coated 100 mm dishes in EBM-2 medium (Lonza, Cat# CC-3156) supplemented with VEGF, IGF-1, EGF, bFGF, hydrocortisone, ascorbate, gentamycin, and 0.5% exosome-depleted FBS (Exo-FBS, Systems Bioscience) at 37 °C and 5% CO_2_. 

#### iPSC

Human peripheral blood mononuclear cells (PBMCs) derived from an individual with no familial history of AD or amyloidosis were reprogrammed to iPSCs using the CytoTune iPSC 2.0 Sendai Reprogramming Kit (Thermo Fisher Scientific). This iPSC line (CW50038 line) was obtained from Coriell Institute and was derived from a cognitively intact, elderly (86 years old), non-Hispanic white female participant with the *APOE 3/3 *genotype. The iPSC line was differentiated into NPCs in accordance with our previously reported approach^[25]^. Cells were plated on day in vitro (DIV) 20 on poly-L-ornithine/laminin plates in D/NPEN medium [Supplementary Table 1] enriched with 20 ng/mL b-NGF, 20 ng/mL NT-3, 20 ng/mL BDNF, and 2 mg/mL DAPT (only from DIV 30 to 34). NPC expansion was transitioned into terminal differentiation of glutamatergic neurons by dissociating with accutase and plating onto six-well plates coated with 100 μg/mL poly-D-lysine (PDL), 20 μg/mL laminin, and 10 μg/mL fibronectin and grown in D/NPEN lacking heparin, and the following small molecules: 20 ng/mL NT3 and 20 ng/mL BDNF. The medium was changed every 2 days or as needed. Duplicate cultures of the cell line were analyzed using IncuCyte Zoom (Sartorius). EVs were used to treat neurons for 24 h to characterize neurite extensions on DIV 51/52.

#### ReN cells

ReNcell CX Human Neural Progenitor Cells were purchased from Millipore (Cat# SCC007). This immortalized human NPC line can be differentiated into neuronal and glial cell lineages. ReN cells were grown on 20 mg/mL purified mouse laminin (Millipore, Cat# CC095)-coated tissue culture plates in ReNcell NSC Maintenance Medium (Millipore, Cat# SCM005) supplemented with 20 ng/mL basic fibroblast growth factor-1 (Millipore, Cat# GF003) and 20 ng/mL epidermal growth factor (Millipore, Cat# GF001) at 37 °C and 5% CO_2_. Cells were used in experiments when they reached 80% confluence, typically 3-4 days after plating. For differentiation experiments, ReN cells were plated in complete medium. The following day, the medium was replaced with fresh medium lacking growth factors. The cells were allowed to differentiate into neurons for 14 days. 

### Transfection of hCMEC/D3

hCMEC/D3 were transfected with the CD9 Cyto-Tracker constructs to label EVs. EVs were labeled with RFP through the expression of pCT-CD9-RFP Cyto-Tracker vector (pCMV, Exosome/Secretory, CD9 Tetraspanin Tag). The cells were transduced using a transfection kit (System Biosciences, Cat# CYTO123-PA-1) and PureFection Transfection Reagent (System Biosciences, Cat# LV750A-1), in accordance with the manufacturer’s protocol. Twenty-four hours after transfection, hCMEC were exposed to 100 nM fluorescent Aβ_40_ HiLyte and the EV-containing supernatant was harvested 48 h later. EVs were isolated from the tissue culture supernatant as outlined below. Once isolated, EVs were pipetted onto glass slides, heat-fixed at 95 °C for 10 min, and fixed again with 100% ethanol for 30 min at 4 °C. Slides were washed three times with PBS and mounted using ProLong Gold Antifade reagent with DAPI (Invitrogen). Specimens were covered with coverslips and fluorescent images [green fluorescence (Aβ HiLyte Alexa Fluor488) and red fluorescence (CD9-RFP)] were acquired using a Nikon Eclipse Ti-U inverted fluorescent microscope. The settings for fluorescence intensity and contrast were optimized and the same settings were used for the analysis of EVs. Intensity plot images were acquired using Nikon-Elements imaging software (NIS).

### Aβ_40_-loaded EVs

EVs were loaded with Aβ_40_ as described earlier. Briefly, hCMEC/D3 cells were grown as a monolayer and split when greater than 90% confluent in exosome-depleted complete medium incubated at 37 °C and 5% CO_2_. Freshly solubilized Aβ_40_ (Anaspec, Cat# AS-20698) or Aβ_40_ HiLyte (Anaspec, Cat# AS-60491-01) was used in the following experiments because this species has been shown to induce proinflammatory reactions in brain microvessels and endothelial cells and is the most abundant of the proamyloidogenic species produced from APP^[26,27]^. Aβ_40_ was dissolved in 0.1 M NH_4_OH basic buffer (Anaspec, Cat# AS-61322) and further diluted in PBS, in accordance with the manufacturer’s instructions. Then, hCMEC/D3 were treated with fresh EBM-2 medium supplemented with growth factors and 100 nM Aβ_40_ for 48 h. This dosage of Aβ_40_ was chosen since the Aβ_40_ levels in AD patients’ CSF are in the nanomolar range. This concentration has also been shown to result in maximal EV loading^[28]^. Control cultures received fresh medium supplemented with PBS as a vehicle control treatment. Treatment was terminated by removal of the medium and EVs were isolated from ~1.7 × 10^7^ cells using the ExoQuick-TC kit (System Biosciences, Cat# EXOTC50A-1). Briefly, 10 mL of hCMEC/D3 culture supernatant was mixed with 2 mL of ExoQuick Exosome precipitation solution. This mixture was incubated overnight at 4 °C to facilitate even coating of the EVs. The next day, the samples were centrifuged at 1500 × *g* for 30 min, the supernatants were removed, and the pellets were centrifuged further at 1500 × *g* for 5 min. The resulting EV pellets were resuspended in PBS for characterization or use in our experiments. The whole fraction of isolated EVs was used in our experiments to account for both small EVs (exosomes) as well as larger particles, both of which can contain Aβ_40_. 

### Nanoparticle tracking analysis

EVs isolated from the supernatant of hCMEC/D3 cells were characterized and analyzed by nanoparticle tracking analysis (NTA) on the Nanosight NS300 (Malvern Instruments Company, Malvern, UK). Optimal detection settings were acquired with the screen gain set to 11 and a detection threshold level of 5 using Nanosight NTA 2.3 Analytical Software (Malvern Instruments Company). This allowed us to track the EV particles in suspension with minimal background [Supplementary Video 1 and Supplementary Video 2]. EV pellets were diluted 100-fold in sterile-filtered PBS and five 15-s frame captures were recorded for each sample. An average of three batches from each group was used to determine the total number of vesicles. EV-Aβ_40_ vesicle number was normalized to the total average number of EV-PBS for all treatments. 

### Enzyme-linked immunosorbent assay

Human Amyloid Beta 40 enzyme-linked immunosorbent assay (ELISA) (Invitrogen, Cat# KHB3481) was used to quantify the levels of Aβ_40_ loaded into EVs. First, EVs were lysed at a 1:1 ratio with RIPA buffer (Thermo Fisher Scientific, Cat# 89900) containing protease inhibitor (Thermo Fisher Scientific, Cat# 78431). The resulting lysate was loaded into a precoated 96-well plate, in accordance with the manufacturer’s instructions, and absorbance was measured using the Colorimetric SpectraMax 190 plate reader and normalized to total protein levels as measured by a BCA assay (Thermo Fisher Scientific, Cat #23223).

### IncuCyte zoom

iPSC-derived NPCs were seeded at a cell density of 40,000 cells per well in a six-well culture plate coated with poly-L-lysine and allowed to differentiate into neurons. Alternatively, ReN cells were seeded at 60,000 cells per well in a 12-well plate coated with laminin. The IncuCyte Zoom automated cell imaging system (Sartorius) was used to automatically acquire kinetic data every hour for either 24 h (iPSC-derived neurons) or 14 days of NPC differentiation (ReN cells) to assess neurite outgrowth and neurogenesis, respectively. iPSC-derived neurons were treated once with EVs, Aβ_40_, or medium alone. Images were taken every hour for 24 h. ReN cells were treated with EVs or Aβ_40_ every other day along with fresh medium over the course of 14 days of differentiation. Neurotrack software was used to quantify neuron cell-body clusters, neurite branch points, and neurite branch points per cell-body area. 

### Quantitative PCR

Total RNA was isolated from cell culture lysates using RNeasy mini kit (Qiagen, Cat# 74104), in accordance with the manufacturer’s instructions. Total nucleic acid concentrations were quantified using the Nanodrop 2000 (Thermo Fisher Scientific). RT-PCR was performed with a total of 100 ng of RNA using the qScript XLT 1-Step RT-qPCR ToughMix Low ROX (Quantabio, Cat# 89236-676) reaction mix and the Applied Biosystems 7500 system (Applied Biosystems, Foster City, CA, USA). TaqMan Gene Expression Assays and the following primers were used: Hs00208382_m1: Mfn2 primer; Hs00250475_m1: Mfn1 primer; Hs00953477_m1: SIRT3 primer; Hs00697394_g1: Mff primer; Hs01047018_m1: OPA1 primer; Hs00608023_m1: Bcl-2 primer; and DNM1L-Hs01552605_m1: Drp-1 primer. Human GAPDH (ThermoFisher Scientific, Cat# 4310884E) was used as an endogenous control for sample normalization. PCR product specificity was determined using melting curve assessment and gene expression differences were determined using the ΔΔCt method.

### Transmission electron microscopy

#### EVs

EV samples from both control and Aβ_40_-treated groups were visualized using transmission electron microscopy (TEM). EV suspensions were fixed in 2% paraformaldehyde for 1 h and then loaded on a copper mesh grid and allowed to incubate for 45 min. The grids were allowed to dry overnight. Then, the EV suspension was stained using 1% uranyl acetate. Once the grids were dry, EVs were imaged using a JEOL JEM-1400 transmission electron microscope with images acquired using an AMT BioSprint digital camera.

#### ReN cells

Cells grown on glass coverslips were fixed overnight at 4 °C in 2% glutaraldehyde in 0.1 M phosphate buffer, before being incubated for 1 h in 2% osmium tetroxide in 0.1 M phosphate buffer, dehydrated through a series of graded ethanol concentrations, and embedded in EM-bed (Electron Microscopy Sciences). The glass coverslips were removed by treatment with hydrofluoric acid. Next, 100 nm sections were cut on a Leica Ultracut EM UC7 ultramicrotome and stained with uranyl acetate and lead citrate. The grids were viewed at 80 kV in a JEOL JEM-1400 transmission electron microscope and images were captured by an AMT BioSprint digital camera. For quantitative analysis, fields were chosen at random, and acquisition and quantification were performed using FIJI (NIH) and GraphPad Prism 9.3.1 software, respectively. Morphometric analyses of the mitochondria and substructures were performed by three investigators in a randomized, double-blind manner, which included taking the images, quantifying the data, and running the statistics separately. Images were obtained at random from three independent experiments^[29]^.

### Western blot

Treated cells were washed with PBS and lysed with radioimmunoprecipitation assay (RIPA) buffer (Thermo Fisher Scientific, Cat# 89900) supplemented with protease inhibitors (Thermo Fisher Scientific, Cat# 78431). Total protein concentration was determined using the BCA protein assay kit (Thermo Fisher Scientific, Cat# 23223). Samples were prepared for immunoblotting with RIPA diluent and sodium dodecyl sulfate (SDS). Then, 15-20 μg of total protein was loaded per lane on 4%-20% midi-PROTEAN TGX Stain-Free polyacrylamide precast gels (Bio-Rad Laboratories, Cat# 4568096) and transferred to a nitrocellulose membrane using the Trans-Blot Turbo Transfer System (Bio-Rad Laboratories, Cat# 1704159). Membranes were blocked using Intercept TBS Blocking Buffer (LICOR, Cat# 927-60001) for 1 h at room temperature. The next day, blots were incubated overnight at 4 °C with primary antibodies [Supplementary Table 2] diluted in 5% bovine serum albumin (BSA). Blots were washed three times using Tris-buffered saline with 0.1% Tween-20 (TBS-T) before incubation with secondary antibody at room temperature for 1 h. Analysis of GAPDH (Novus Biologicals, Cat# NB600-502FR or Cat# NB600-5021) was performed as an internal control and for sample normalization. Bands were imaged with the Odyssey CLX Imaging System (LICOR) and signal quantification was performed with Image Studio 4.0 software (LICOR).

### Seahorse XF analyzer

Oxygen consumption rate (OCR) and extracellular acidification rate (ECAR) were measured on a Seahorse XF24 Analyzer (Agilent Technologies, Inc.) using the Seahorse Mito Stress Test kit (Agilent Technologies, Inc., Cat# 103015-100). ReN cells were seeded at a density of 40,000 cells per well in a 24-well Seahorse tissue culture microplate (Agilent Technologies, Inc., Cat# 100777-004) coated with purified mouse laminin (Millipore, Cat# CC095-5MG). Once cells reached ~80% confluence, they were treated with EV-PBS, EV-Aβ_40_, or Aβ_40_ alone for 24 h. Three basal respiratory measurements were obtained before injections of oligomycin (1.5 mM), FCCP (0.5 mM), and rotenone/antimycin A (0.5 mM). OCR and ECAR measurement results were then recorded, and all data were normalized to total protein levels (mg/mL) per well as measured by a BCA assay. Each data point represents the average measurement from five separate wells.

### Confocal microscopy

NPCs were cultured on laminin-coated eight-well chamber slides (Ibidi, Cat# 80841) and treated with EV-PBS, EV-Aβ_40_, or Aβ_40_ for 24 h. The cells were subsequently fixed with 4% PFA for 10 min, washed with PBS, and permeabilized with PBS containing 0.1% Triton X-100 (Millipore Sigma, Cat# 93443). The fixed and permeabilized cells were incubated with blocking buffer (20% FBS and 0.5% Tween-20 in PBS) for 1 h. Then, samples were incubated overnight at 4 °C with primary antibodies [Supplementary Table 3]. The next day, primary antibody was aspirated, and slides were washed with PBS and incubated with Alexa Fluor-conjugated secondary antibodies for 2 h at room temperature. Slides were mounted with a glass coverslip in Fluoromount-G Mounting Medium with 4’,6-diamidino-2-phenylindole (DAPI; Invitrogen, Cat# 00-4959-52) to visualize the nuclei. Immunofluorescent images were captured with a Fluoview 1200 confocal microscope (Olympus, 20× dry lens, or 40× or 100× oil immersion lens).

For the nuclear factor-κB (NFκB) measurements, mean fluorescence intensity was measured in the nuclear areas outlined by DAPI staining using FIJI software (v.2.3.0, NIH) and the Isodata algorithm. It was then used to convert the NFκB image to a binary mask that included all fluorescence data above the background. Nuclear and whole-cell histogram data were calculated and normalized for the total number of nuclei included in each image. A comparison was then made from the difference in staining intensities. NFκB nuclear translocation is represented by an increase in nuclear:cytoplasmic ratio of signal intensity. For TOM20 measurements, the total area of fluorescence intensity on the acquired images was normalized to the number of nuclei. Confocal microscopy images were analyzed using FIJI software (v.2.3.0, NIH). Images were pre-processed using the Bio-Formats (Available from: https://github.com/ome/bioformats) plug-in splitting fluorescent channels: DAPI (408 nm) and TOM20 (488 nm). Mitochondrial network parameters including mitochondrial footprint, mean branch length, total branch length, and mitochondrial network were analyzed using the Stuart Lab Mitochondrial Network Analysis (MiNA) (Available from: https://github.com/StuartLab) plug-in^[30]^.

### Statistical analysis

Data are presented as the mean ± standard error of the mean (SEM). All statistical tests were performed and all graphs were constructed using GraphPad Prism software 9.3.1. Statistical comparisons were conducted using one-way ANOVA with Tukey’s multiple comparisons test, or an unpaired t-test where indicated. Levels of significance were set to *P < 0.05, **P < 0.01, ***P < 0.001, and ****P < 0.0001.

## RESULTS

### Characterization of brain endothelium-derived EVs loaded with Aβ_40_

**Figure 1:**
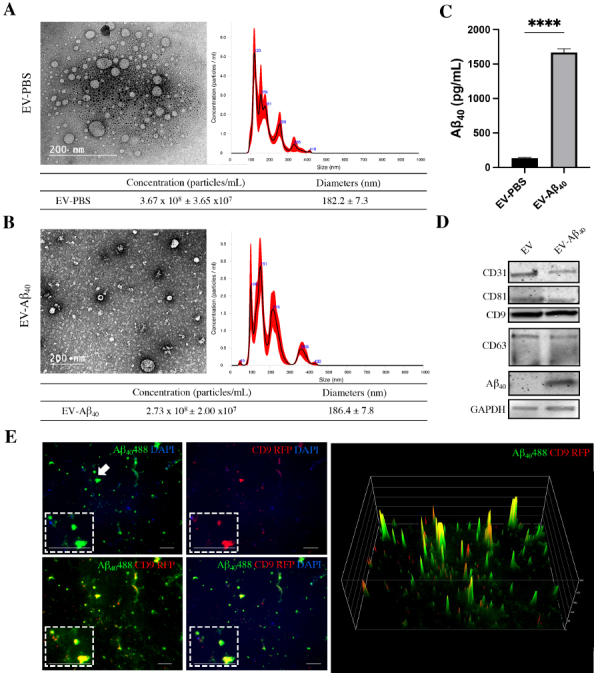
Characterization of brain endothelium-derived extracellular vesicles. Vesicles were isolated from endothelial cell cultures treated with Aβ_40_ or PBS. TEM demonstrates average size and spherical morphology of (A) control EVs and (B) EV-Aβ_40_. NTA of isolated EVs loaded with (A) PBS as a vehicle control and (B) Aβ_40_ shows variable diameter distribution in the nanometer range. Red outlines indicate error bars (± SEM) from five 15-s videos of NTA. (C) ELISA for Aβ_40_ indicates effective enrichment of EVs isolated from medium of endothelial cells treated with Aβ_40_. Student’s unpaired *t*-test, ****P < 0.0001 (isolated vesicle lysates from individual cell culture dishes; *n *= 3). (D) Western blot of lysed EVs for tetraspanin markers CD9, CD63, CD81, and Aβ_40_. CD31 expression confirmed that the EVs were secreted from an endothelial cell line. (E) Fluorescent images of EVs isolated from endothelial cells transfected with CD9-RFP (red) and exposed to fluorescent Aβ_40_ (green). DAPI indicates genetic material (blue). Fluorescent intensity plot shows co-localization of CD9 vesicle marker (red) and Aβ_40_ (green) in the EV-Aβ_40_ group. Scale bar for representative images is 10 µm and zoom inserts are 30 µm. White arrow indicates the region of interest for the zoom inserts. TEM: Transmission electron microscopy; EV: extracellular vesicles; NTA: nanoparticle tracking analysis; SEM: standard error of the mean; ELISA: enzyme-linked immunosorbent assay.

The BBB has been shown to act as a route of entry for Aβ_40_ to transfer into the brain at concentrations in the nanomolar range. Therefore, our studies examined these physiological concentrations using Aβ_40_-loaded EVs. In the initial series of experiments, EVs were isolated from the cell culture medium collected from brain endothelial cells (hCMEC) that had been treated for 48 h with exogenous Aβ_40_ or PBS as a vehicle control. The isolated EVs were assessed by nanoparticle tracking analysis (NTA) and transmission electron microscopy (TEM) to characterize EV size, morphology, and distribution. These analyses showed that the isolation procedure yielded a heterogeneous range of sizes of EVs isolated from both control [Figure 1A] and Aβ_40_-treated [Figure 1B] endothelial cells. Most EVs fell between 100 and 400 nm in size for each group. EVs isolated from control hCMEC had a mean size of 182.2 nm (±7.3 nm) and a total mean EV concentration of 3.67 × 10^8^ particles/mL. EVs isolated from hCMEC treated with Aβ_40_ had a mean size of 186.4 (±7.8 nm) and a total mean EV concentration of 2.73 × 10^8^ particles/mL [Figure 1A and B]. We did not observe any significant change in particle size after treatment with Aβ_40_. TEM analysis of the isolated EVs confirmed the size and morphology of control EVs and EV-Aβ_40_ (Figure 1A and B, respectively). EVs isolated from hCMEC treated with Aβ_40_ exhibited effective packaging of Aβ_40_ comparable to that of those isolated from PBS-treated cells, as measured by ELISA [Figure 1C]. Once the size distribution was determined, we analyzed EVs for the presence of specific markers, as well as the presence or absence of Aβ_40_ in whole-vesicle lysates. Ubiquitous vesicular markers - CD9, CD63, and CD81 - are tetraspanin proteins present in lipid membrane vesicles of various sizes. All three markers were expressed in isolated EVs, along with Aβ_40_ in EVs isolated from Aβ_40_-treated cells [Figure 1D]. Additionally, we probed for CD31 to confirm that our EVs were derived from endothelial cells making up the BBB. Finally, hCMEC were transfected with CD9-RFP, followed by treatment with green fluorescent Aβ_40_, to visualize co-localization of the EV marker CD9. Uptake of the CD9-RFP and co-localization with the green fluorescence signal from Aβ_40_ HiLyte were assessed by fluorescence microscopy. A surface intensity plot was then created to visualize the intensity peaks of both green (Aβ_40_) and red (CD9) signals and their co-localization. Partial overlap of the two signals demonstrated the diversity of EV species and the efficacy of Aβ_40_ uptake into these vesicles [Figure 1E]. In addition, the EVs stained positive for DAPI, suggesting the uptake of genetic material into them. It is expected that there is a heterogeneous mixture of free Aβ_40_ (partially due to EV rupture during the preparation and the efficacy of Aβ_40_ uptake) as well as EV-associated Aβ_40_ in these isolates. 

### NPCs are vulnerable to aberrant neurogenesis when treated with brain endothelium-derived EVs containing Aβ_40_

**Figure 2:**
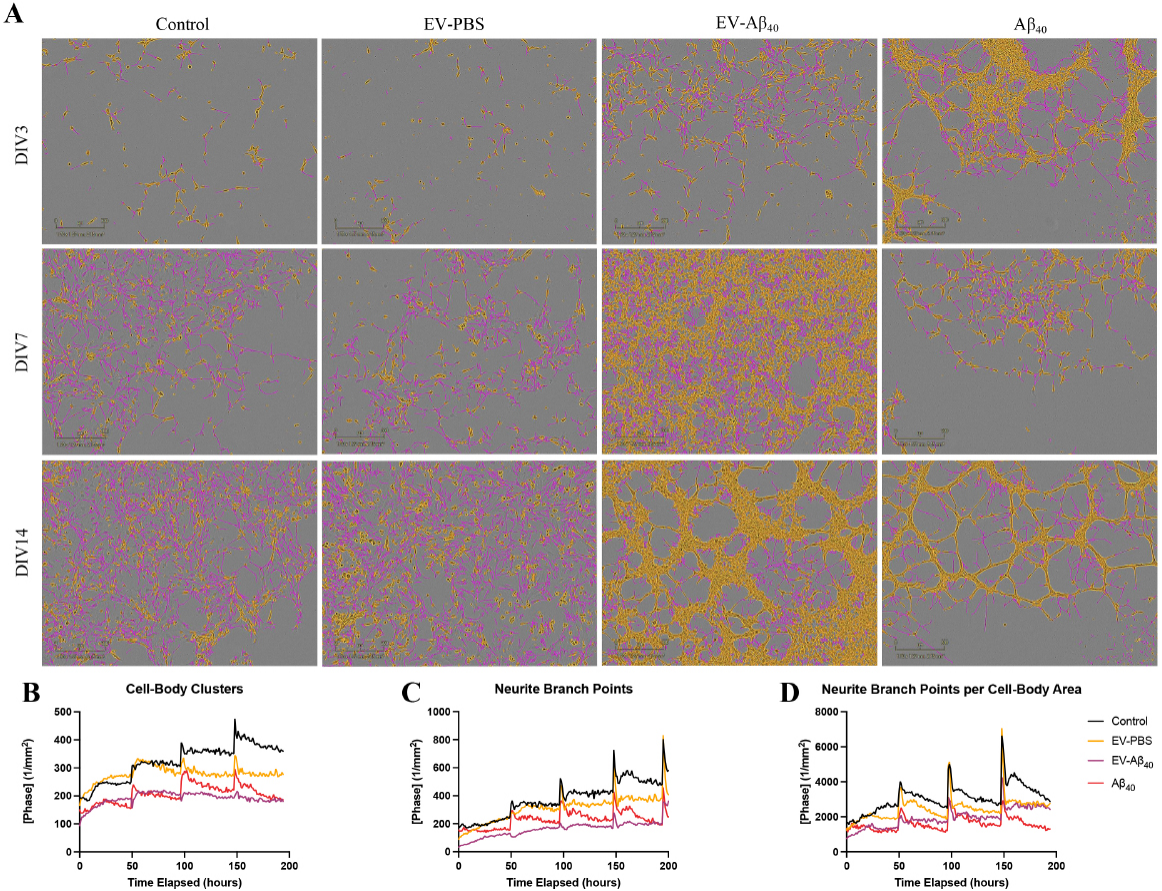
EV-Aβ_40_ induce aberrant NPC differentiation. (A) IncuCyte Zoom analysis of NPC neurogenesis. ReN cells were exposed to EV-PBS, EV-Aβ_40_, or Aβ_40_ every other day for 14 days of differentiation. Representative images from DIV3, DIV7, and DIV14 were taken where pink indicates neurite extensions and yellow represents cell bodies. Quantification of IncuCyte images demonstrates the change in rate of (B) total cell-body cluster area, (C) total neurite branch points, and (D) the ratio of neurite branch points to cell-body area. Graphs represent the average of individual pictures taken in nine image grids over 14 days. Additionally, DIV3 corresponds to timepoint 0 on the graph, the first instance when images were taken of cells after they were plated and treated. EV: Extracellular vesicles; NPC: neural progenitor cell.

In the next series of experiments, we assessed the impact of EVs on NPC differentiation using ReN cells. The ReN-derived NPCs were allowed to differentiate into neurons through the removal of growth factors from the medium, and images were taken every hour over 14 days. Treatment with EV-Aβ_40_ resulted in lower ratios of neurite extension (traced in pink) to cell body (traced in yellow), compared with those of NPCs exposed to control EVs, which displayed more healthy dendritic extensions and synapse formation between cells [Figure 2A]. Cell-body clusters, outlined in yellow, tended to form dense clusters in the EV-Aβ_40_ and Aβ_40_ groups compared with those in the untreated and EV-PBS NPCs [Figure 2B]. The total number of neurite branch points was calculated as an indicator of proper development and synapse formation. In addition to a higher tendency for cell bodies to cluster together, both Aβ_40_ groups (EV-Aβ_40_ and Aβ_40_ alone) showed reduced numbers of branch points (indicated by pink tracing of the neurites). The number of branch points/cell-body area tended to be higher in control and EV-PBS groups than in EV-Aβ_40_- and Aβ_40_-treated ReN-derived NPCs [Figure 2C]. A decrease in the neurite branch points to cell-body ratio was observed during this differentiation time course following treatment with Aβ_40_. Meanwhile, the control groups displayed a tendency for an elevated growth rate [Figure 2D]. Upon treatment with EVs and fresh medium, it was common to observe elevated rates of growth upon IncuCyte analysis (spikes in plots B-D), with cells returning to a basal state of growth in the time following replacement of the medium.

We also confirmed the impact of EV-Aβ_40_ on decreased neurite growth in an iPSC-derived stem cell model. The experiments were based on a human-derived iPSC line that was sequentially differentiated into NPCs and neurons for *in vitro *neurite extension studies. Cortical neurons were differentiated to 50 DIV and assessed by immunocytochemistry for neuron-specific markers (NeuN and β-tubulin III) [Supplementary Figure 1A]. At DIV 50, neurons were treated with EV-PBS or EV-Aβ_40_ for 24 h. Following this treatment, images were taken on the IncuCyte every hour for 6 h to determine the effect of EV-associated Aβ_40_ on neurite length. Similar to the results seen with the ReN-derived NPCs, the iPSC-derived neurons showed a reduction in the ratio of neurite length to cell-body area [Supplementary Figure 1B and C].

### Brain endothelium-derived EVs containing Aβ_40_ alter mitochondrial crista morphology

**Figure 3:**
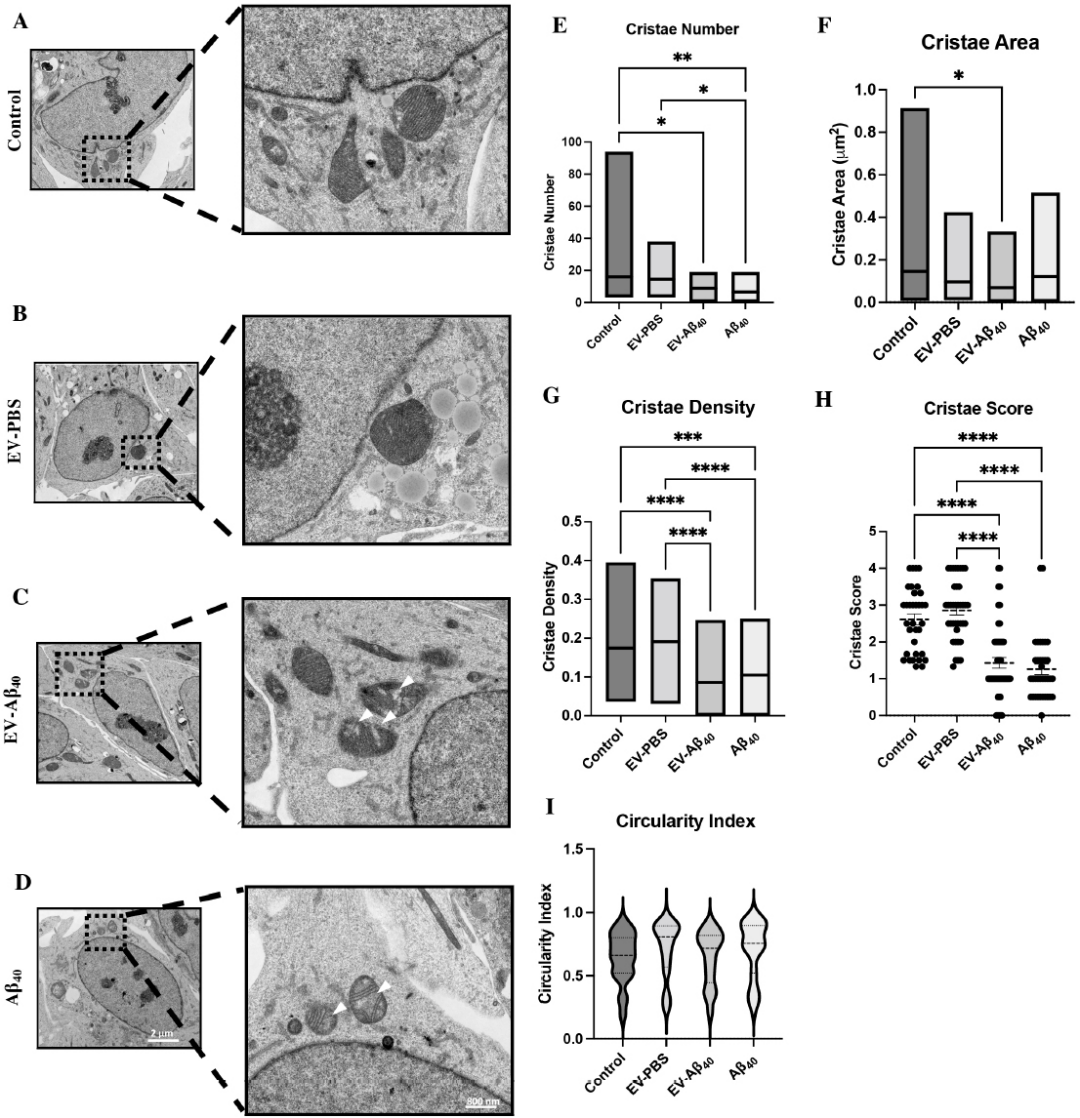
EV-Aβ_40_ alter the morphology of NPC mitochondrial cristae. Representative TEM images of morphology of perinuclear mitochondria and cristae in (A) untreated NPCs or those treated with (B) EV-PBS, (C) EV-Aβ_40_, or (D) Aβ_40_ alone as a positive control for 24 h. Quantification of (E) crista number, (F) crista surface area, (G) crista density, (H) crista score, and (I) circularity index. Scale bar for representative images is 2 µm and zoom inserts are 800 nm. *P < 0.05, **P < 0.01, ***P < 0.001, and ****P < 0.0001. *n *= 30 images per group and 38-72 mitochondria per group. EV: Extracellular vesicles; NPC: neural progenitor cell; TEM: transmission electron microscopy.

To investigate the cause of NPC disruption and aberrant neurogenesis upon exposure to EV-Aβ_40_, we sought to understand whether metabolic regulation of NPCs was being affected through alterations in mitochondrial functionality. Mitochondria are essential organelles that mediate cellular function and survival. Organs and tissues that have high energetic requirements, such as the brain, have higher overall levels of mitochondria. The morphology of mitochondria in NPCs was analyzed using TEM following 24 h of treatment with medium alone (control), EV-A_40_, control EVs (EV-PBS), or A_40_ alone. Untreated NPCs and NPCs exposed to EV-PBS exhibited healthy mitochondria with dense, regular, inner mitochondrial membrane formation [Figure 3A and B]. Meanwhile, NPCs exposed to EV-Aβ_40_ and Aβ_40_ alone exhibited morphological changes in crista structures [Figure 3C and D]. In addition, the presence of white patches, or crista “voids” (indicated by the arrows), was consistently seen in both EV-Aβ_40_- and Aβ_40_-exposed groups, but not in the control groups (control and EV-PBS).

The mitochondrial area and crista structures were analyzed in the perinuclear region of NPCs, as previously described^[29]^. Limiting the analysis to this region is crucial to control for the high rate of mitochondrial dynamics normally found in the growth extension regions of differentiating cells. The total perinuclear mitochondrial area was not significantly changed between groups (data not shown). Using TEM analysis, we observed a significant decrease in crista number in both EV-Aβ_40_- and Aβ_40_-exposed NPCs compared with the level in untreated or EV-PBS-exposed cells [Figure 3E]. The total crista area was determined from individual mitochondria and the summation of outlined crista surface area was measured. The surface area of perinuclear mitochondrial cristae was significantly decreased between control NPCs compared with that of EV-Aβ_40_-exposed cells [Figure 3F]. In addition, we measured the density of cristae, which reflects the ratio of crista surface area to the associated mitochondrion area. The relative density of cristae exhibited a significant decrease in both Aβ_40_ treatment groups (EV-Aβ_40_ and Aβ_40_ alone) compared with the level in the control groups [Figure 3G]. Similarly, mitochondria in each group were assigned morphometric crista scores to visually assess crista definition. This scale ranges from 0, corresponding to no sharply defined cristae, to 4, corresponding to many regular cristae. Treatment with EV-Aβ_40_ or Aβ_40_ alone resulted in a significantly lower crista score than in the control groups. This suggests that exposure to Aβ_40_ results in the adoption of irregular shapes [Figure 3H]. Circularity index is a relative measure of mitochondrial shape, with 1.0 representing a complete sphere and 0.0 representing an extreme elliptical or elongated shape. NPCs treated with EV-Aβ_40_ tended to display a greater fluctuation between spherical and elongated extremes than in the control treatments [Figure 3I].

### Mitochondrial footprint alterations in NPCs exposed to EVs containing Aβ_40_

**Figure 4:**
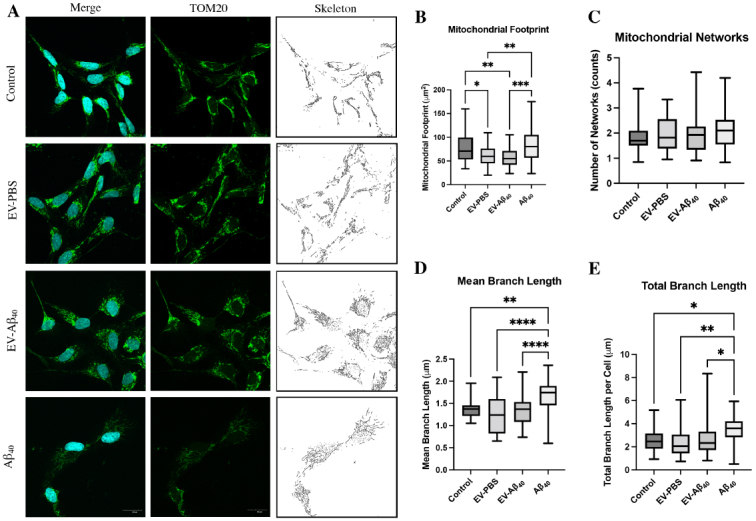
Analysis of the mitochondrial network skeletons in NPCs using MiNA. (A) Confocal images of NPCs stained with DAPI (blue) and TOM20 (green) for tracking the mitochondrial membrane. MiNA plug-in on ImageJ was used to skeletonize the mitochondria. Box and whisker plots quantify the (B) mitochondrial footprint, (C) total mitochondrial networks, (D) mean branch length, and (E) total branch length. Box plots show the mean (horizontal line), first to third quartile (box), and the extreme values within the 95% confidence interval of the interquartile range (whiskers). Scale bar for representative images is 20 µm. *P < 0.05, **P < 0.01, ***P < 0.001, and ****P < 0.0001, using one-way ANOVA. *n *= 6-9 images per group, each image containing 3-12 NPCs. EV: Extracellular vesicles; NPCs: neural progenitor cells; MiNA: mitochondrial network analysis; DAPI: 4’,6-diamidino-2-phenylindole.

To further explore the mitochondrial dynamic changes in NPCs exposed to EV-Aβ_40_ or Aβ_40_ alone, we next focused on morphological features of mitochondrial networks. TOM20, a translocase protein located on the outer mitochondrial membrane, was used to stain mitochondrial networks within treated NPCs [Figure 4A]. Using MiNA, we quantified the networks using a skeletonized overlay approach. Mitochondrial footprint, which is the total area of the image with a signal, was reduced in both vesicle groups (EV-PBS and EV-Aβ_40_) compared with that in untreated cells; however, this impact was more pronounced in NPCs exposed to EV-Aβ_40_ [Figure 4B]. Interestingly, the overall mitochondrial network number, a measure of networks comprising more than one branch, was unchanged among all groups [Figure 4C]. While networks remained unchanged, network fusion, or the restricted ability of mitochondria to divide, was apparent in Aβ_40_-treated NPCs, indicating possible mitochondrial disruption. This was evident by enhanced fragmentation of larger networks in the Aβ_40_ group, resulting in longer branch lengths, compared with fragmentation of slightly smaller relative networks in control NPCs [Figure 4D]. Additionally, the presence of long branches [Figure 4E] and a larger network size may indicate events leading to the occurrence of hyperfusion and the potential for many of the mitochondria to exhibit an elongated branched network. Hyperfusion can also be induced by stress or mitophagy and can decrease the ability of the cell to divide and differentiate properly. An increase in total overall branch length [Figure 4E] may be related to this phenomenon when fission machinery is disrupted or inhibited as a compensatory mechanism. Overall, our mitochondrial tracking using TOM20 demonstrated that the exposure of NPCs to Aβ_40_ (either alone or in the form of EV-Aβ_40_) results in morphological changes to the mitochondria, as well as alterations in the organelle structure that lead to possible dynamic shifts within the cell.

### EV-Aβ_40_ contribute to the dysregulation of mitochondria by impairing metabolic respiration

**Figure 5:**
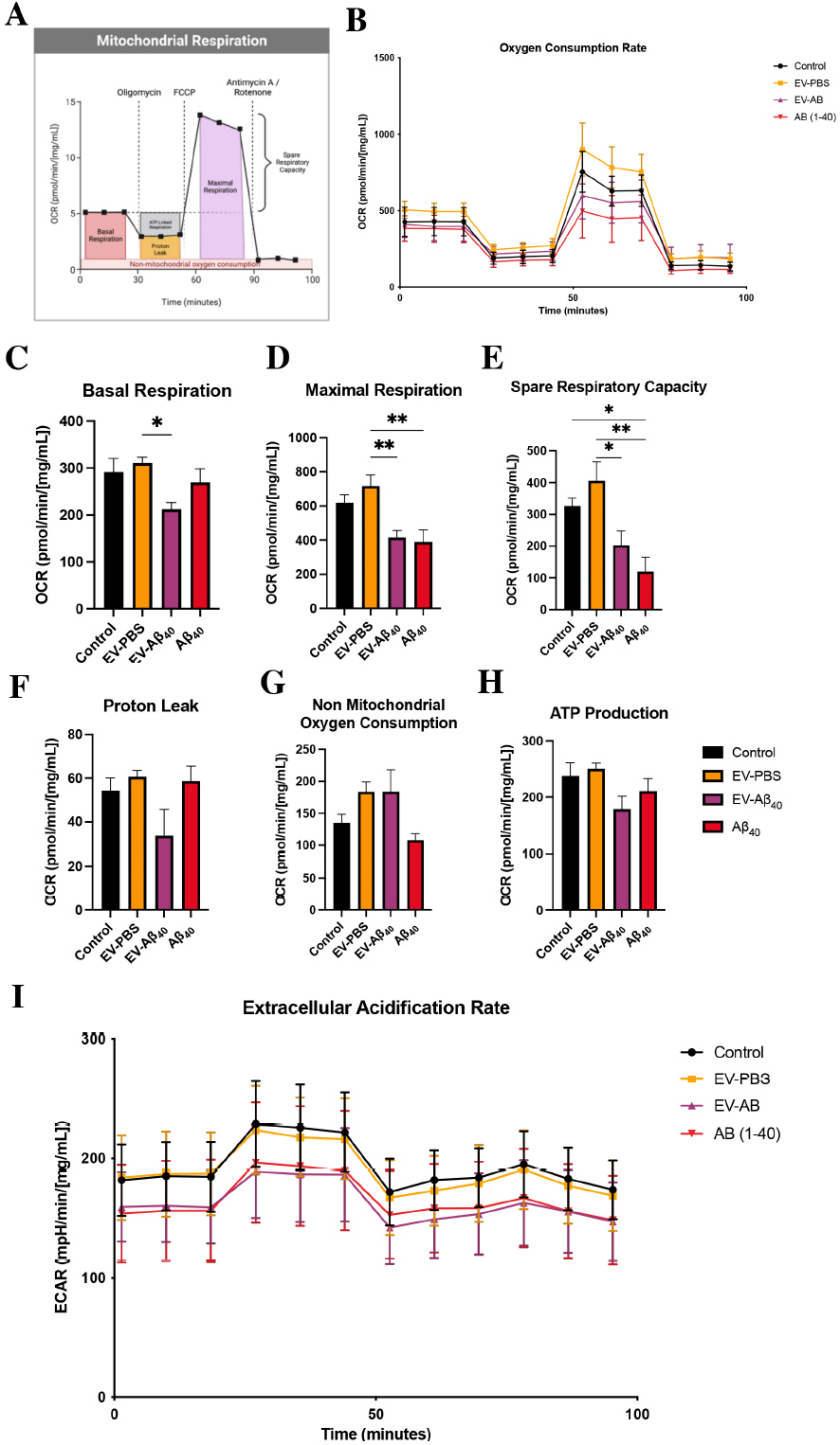
Impact of EV-Aβ_40_ on NPC mitochondrial respiratory function. (A) Schematic of the mitochondrial stress test assay. (B) OCR in untreated NPCs and NPCs exposed to EV-PBS, EV-Aβ_40_, or Aβ_40_ alone for 24 h and normalized to protein levels. Respiratory measurements of OCR associated with (C) basal rate, (D) maximal respiration, (E) spare respiratory capacity, (F) proton leakage, (G) non-mitochondrial oxygen consumption, and (H) ATP production. (I) Glycolysis is measured by ECAR in the same cells as (B-H). All graphs present the mean ± SEM. Statistical analysis was performed using one-way ANOVA, *P < 0.05, **P < 0.01, and ***P < 0.001, *n *= 5 per group. EV: Extracellular vesicles; NPCs: neural progenitor cells; OCR: oxygen consumption rate; ECAR: extracellular acidification rate; SEM: standard error of the mean.

After confirming differential regulation in mitochondrial footprint and branch length dynamics, we sought to evaluate whether these effects, combined with changes in the morphology of cristae, altered the respiratory capacity of NPCs. Healthy differentiating NPCs rely mainly on mitochondrial respiration and maintain glycolysis at low levels. Therefore, we analyzed whether EV-Aβ_40_ treatment could alter mitochondrial respiration and subsequent ATP production using Seahorse technology. First, we assessed oxygen consumption rate (OCR) as an indicator of mitochondrial respiration [Figure 5A and B]. Treatment with Aβ_40_, either alone or in the form of EV-Aβ_40_, resulted in a decrease in the compensatory rate of respiration compared with that of untreated NPCs or treatment with control EVs. In the subsequent analyses, OCR was measured following injections with oligomycin, to inhibit complex V (ATP synthase) and force the cell into compensatory mechanisms for ATP production; FCCP, to disrupt membrane potential and uncouple the proton gradient across the inner membrane; and rotenone/antimycin A, to block complexes I and III, which eliminates electron transport chain (ETC) mitochondrial respiration. NPCs treated with EV-Aβ_40_ had significantly lower basal respiration [Figure 5C], maximal respiration [Figure 5D], and spare respiratory capacity [Figure 5E] than untreated NPCs or EV-PBS-treated cells. These parameters measure the capacity of the cells to respond to energy demands and their ability to increase respiration. Proton leakage is indicated by the remaining basal respiration not coupled to ATP production, which showed no statistically significant differences among any of the treatment groups [Figure 5F]. In contrast, both vesicle-treated groups (EV-PBS and EV-Aβ_40_) appeared to exhibit an increased ability to respire by non-mitochondrial cellular enzymes that consume oxygen [Figure 5G]. ATP production after treatment with EV-Aβ_40_ exhibited a tendency to decrease compared with that in the controls [Figure 5H]. We also assessed the extracellular acidification rate (ECAR) as a measure of cellular glycolysis. ECAR values demonstrated a tendency to decrease in NPCs exposed to EV-Aβ_40_ or Aβ_40_ alone relative to the levels in control groups [Figure 5I].

### EV-Aβ_40_ dysregulate mitochondrial fusion and fission machinery

**Figure 6:**
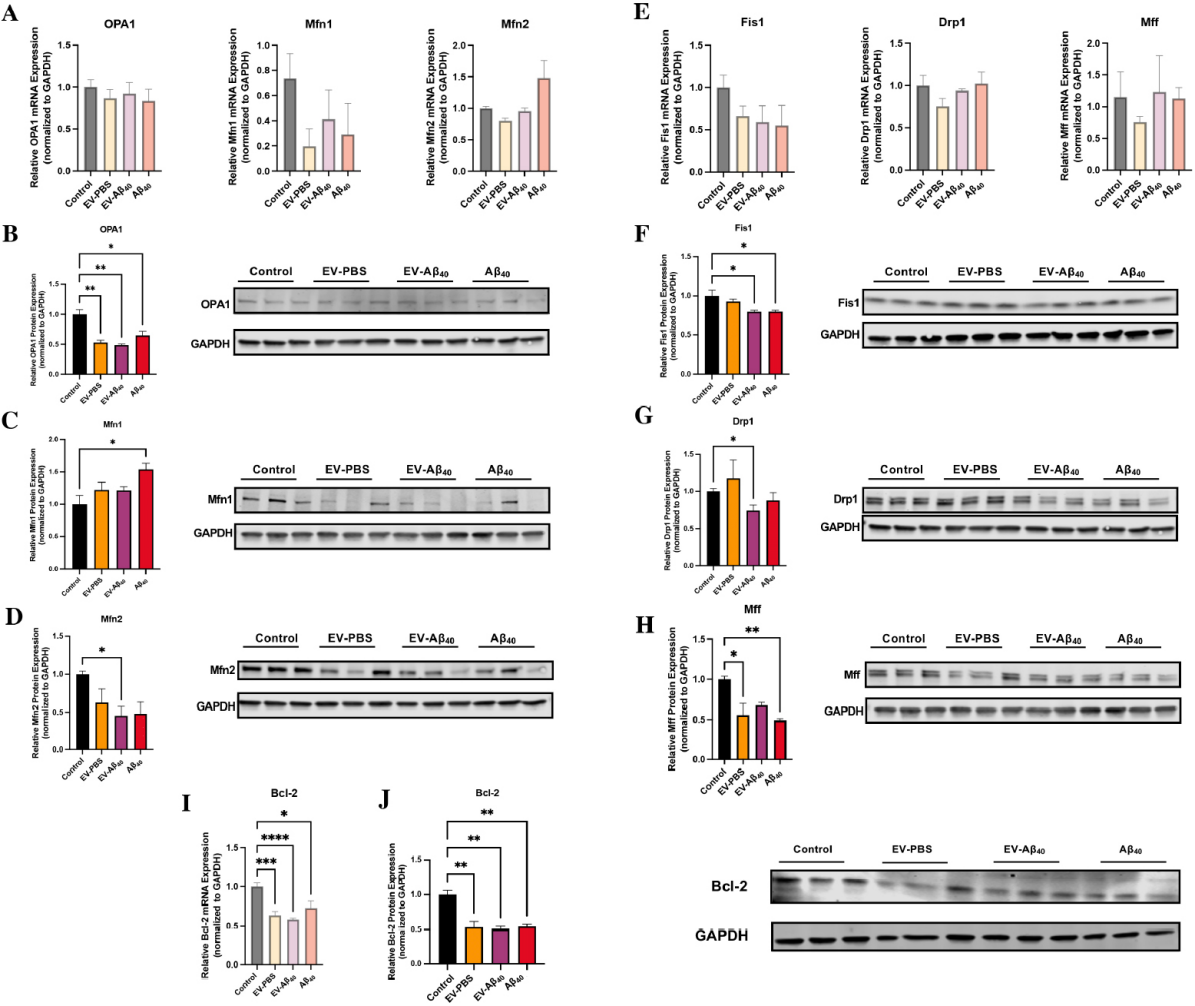
NPC exposure to EV-Aβ_40_ causes dysregulation of mitochondrial fusion and fission machinery. (A) Total mRNA expression was assessed for fusion modulators: *OPA1*, *Mfn1*, and *Mfn2*. The total protein expression levels were assessed along with representative western blots for fusion proteins (B) OPA1, (C) Mfn1, and (D) Mfn2. GAPDH was used as a housekeeping control for all experiments. Additionally, modulators of fission, *Fis1*, *Drp1*, and *Mff*, were assessed via qPCR (E). Total protein expression and representative western blots for mitochondrial fission proteins (F) Mff, (G) Drp1, and (H) Fis1 were quantified. Bcl-2 was probed for (I) total mRNA gene expression and (J) total protein expression by western blotting. Bar graphs indicate mean ± SEM. Statistical analysis was performed using one-way ANOVA, *P < 0.05, **P < 0.01, ***P < 0.001, and ****P < 0.0001, *n *= 3-6 independent experiments per group. EV: Extracellular vesicles; NPC: neural progenitor cell; SEM: standard error of the mean.

Mitochondrial morphology is crucial for cellular health and normal metabolic function. To better understand the main functions that regulate mitochondrial morphology, mitochondrial fusion and fission processes were assessed after treatment with EVs or Aβ_40_. Mitochondrial fusion is mainly regulated by proteins of the outer mitochondrial membrane, mitofusin 1 (Mfn1) and mitofusin 2 (Mfn2), and of the inner mitochondrial membrane, optic atrophy protein (OPA1). We assessed the expression of Mfn1, Mfn2, and OPA1 through mRNA and total protein expression. Although no significant change in total mRNA expression was found among the studied fusion-related genes [Figure 6A], differential protein expression was observed. Specifically, there was a significant decrease in the expression of OPA1 and Mfn2 proteins in NPCs exposed to EV-Aβ_40_, while Mfn1 showed a modest but not statistically significant increase in the cells treated with Aβ_40_ alone [Figure 6B-D].

In addition to mitochondrial fusion, fission proteins are essential to mitochondrial maintenance. They include mitochondrial fission factor (Mff), dynamin-related protein 1 (Drp1), and mitochondrial fission protein 1 (Fis1). Similar to the results of the fusion genes, the expression of the fission genes showed no significant differences across the experimental groups [Figure 6E]. Nevertheless, fission proteins were differentially expressed between the treatment groups. Fis1, a common receptor for Drp1 to promote mitochondrial division, was significantly decreased in both Aβ_40_ groups (EV-Aβ_40_ and Aβ_40_ alone) [Figure 6F]. In addition, Drp1 protein expression was found to be decreased in EV-Aβ_40_-treated NPCs [Figure 6G]. Expression of Mff protein, which is an important regulator in the recruitment of Drp1 to the mitochondria, was also significantly reduced after treatment with Aβ_40_ and tended to show lower levels in EV-Aβ_40_ [Figure 6H]. This overall decrease in fission regulators supports our findings in mitochondrial morphology that indicated a possible hyperfused state in which mitochondrial division is impaired, leading to a shift in the dynamic balance that results in cell stress and metabolic dysfunction. 

Mitochondria are also key regulators of the apoptotic pathway. This involves Bcl-2, a modulator suppressing apoptosis and regulating cell death by mediating mitochondrial membrane permeability. To assess *Bcl-2 *levels, total mRNA levels were assessed by qPCR and showed a significant decrease in NPCs exposed to all of the treatments (EV-PBS, EV-Aβ_40_, or Aβ_40_ alone) compared with that of the untreated control [Figure 6I]. Consistent with this, total protein expression for Bcl-2 was significantly lower in all experimental groups than in untreated NPCs [Figure 6J]. Taken together, our data suggest that an imbalance in normal fusion and fission processes occurs in NPCs exposed to EV-Aβ_40_ or Aβ_40_ alone. This imbalance may lead to the remodeling of cristae, as shown in Figure 3, and further apoptosis-associated responses in the mitochondria.

### Diminished SIRT3 expression and activation of NFκB in NPCs exposed to EV-Aβ_40_

**Figure 7:**
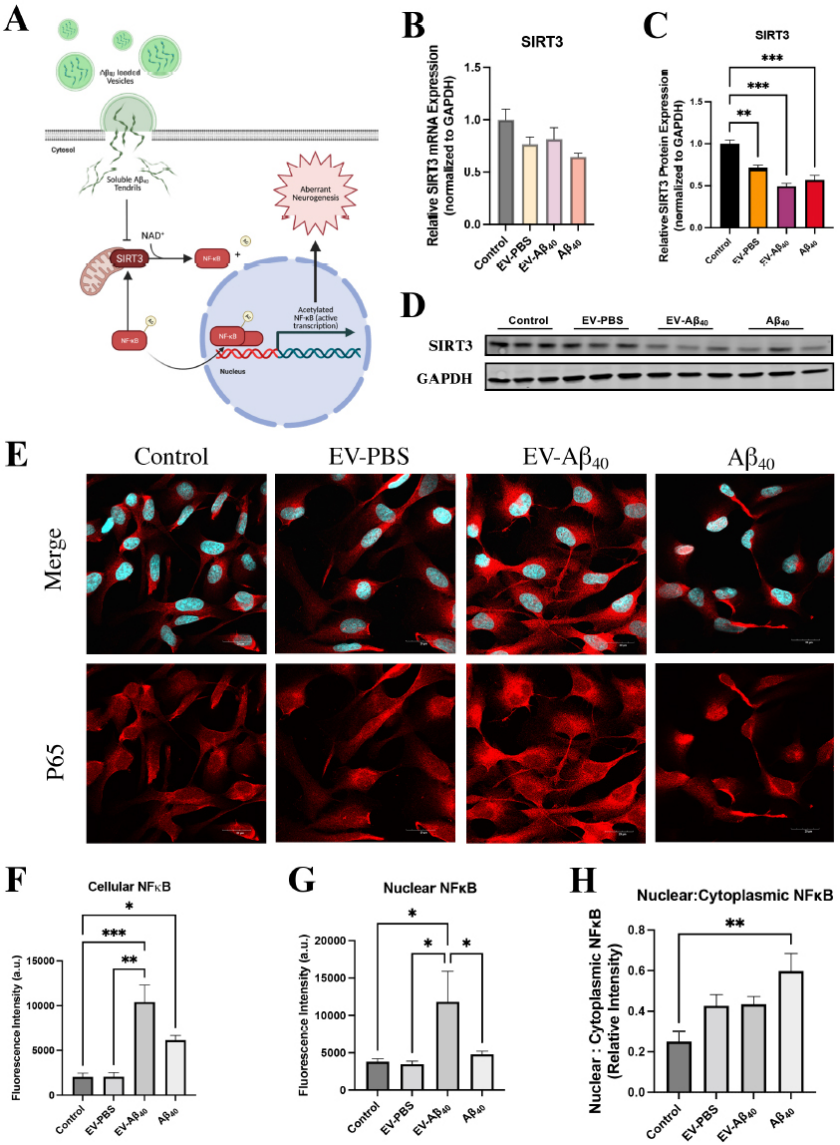
Expression of SIRT3 and NFκB is affected upon exposure to Aβ_40_. (A) Schematic of mitochondrially dependent SIRT3 function affecting NFκB activation in NPCs. Reduced SIRT3 expression inhibits the deacetylation of NFκB, which allows the acetylated form to translocate into the nucleus and induce downstream inflammatory responses. SIRT3 expression was probed for both (B) total mRNA expression and (C) total protein expression. (D) Representative western blots. (E) Confocal images of NPCs after 24 h of treatment stained for DAPI (blue) and p65 protein (red) indicate translocation of NFκB into the nucleus of cells in the Aβ_40_ groups. Images were further quantified for (F) total NFκB cell fluorescence, (G) nuclear NFκB, and (H) the nuclear-to-cytoplasmic ratio of NFκB fluorescence. Bar graphs indicate mean ± SEM. Statistical analysis was performed using one-way ANOVA, *P < 0.05, **P < 0.01, ***P < 0.001, and ****P < 0.0001, *n *= 3 independent experiments per group. EV: Extracellular vesicles; NPC: neural progenitor cell; DAPI: 4’,6-diamidino-2-phenylindole; SEM: standard error of the mean.

To assess the downstream pathways that may alter mitochondrial dynamics in NPCs, we probed for sirtuin 3 (SIRT3) expression, a mitochondrially specific NAD-dependent deacetylase. One of the prominent targets of SIRT3-mediated deacetylation is NFκB [Figure 7A]. *SIRT3 *gene expression was assessed by qPCR, which showed no significant differences across any of the treatment groups [Figure 7B]. However, there was a marked decrease in SIRT3 protein expression in NPCs exposed to EV-Aβ_40_ and Aβ_40_ alone [Figure 7C and D].

We then stained NPCs to examine the expression levels of p65, a subunit of the NFκB heterodimer that is needed for nuclear translocation [Figure 7E]. We assessed the levels of NFκB in the nucleus as an indicator of activation and demonstrated that the group treated with EV-Aβ_40_ had increased expression of NFκB in the nucleus compared with the levels in other treated groups, as quantified by total fluorescence intensity [Figure 7F] and the relative intensity of nuclear staining [Figure 7G]. We also assessed the ratio of p65 fluorescence in the nucleus compared to that in the cytoplasm [Figure 7H]. This analysis indicated that NPCs treated with Aβ_40_ alone exhibited the most significant translocation of NFκB into the nucleus, while the EV groups showed a trend towards higher nuclear-to-cytoplasmic translocation ratios. It is important to consider that the relative nuclear-to-cytoplasmic ratio in the EV-Aβ_40_ group might be affected by aberrant neurogenesis that also occurs in this group.

## DISCUSSION

Unbalanced oxidation and oxidative stress have been widely postulated to be common factors that contribute to pathological aging and aberrant neurogenesis of NPCs. Aβ_40_ has also been shown to possibly impair the viability and differentiation of NPCs by disrupting mitochondrial signaling^[31,32]^. The significance of these events is related to the fact that dysfunctional mitochondria are the main inducers of oxidative stress and imbalance of the redox status, which are the critical factors in the stimulation of immune activation, neurotoxicity, and aberrant NPC proliferation^[33-37]^. Additionally, EVs have recently been postulated to be significantly involved in various neurodegenerative diseases, including Aβ pathology^[16,17]^. One study has demonstrated that Aβ_40_ can be packaged into EVs and shed into the brain microenvironment, and that human Aβ_40_ plaques are enriched with EV proteins^[9]^. The implications of endogenous Aβ_40_ and Aβ_40_ trafficked into the brain may translate into clinical manifestations of AD, such as dementia, and neurodegeneration. Because neurogenesis has been accepted to occur in the adult brain, it is also a crucial modulator of brain plasticity, repair, as well as learning and memory functions^[24,38]^. Neurogenesis in the adult brain occurs in distinct zones from NPCs that reside in several parts of the brain, including the subgranular zone (SGZ) and the dentate gyrus (DG) in the hippocampus^[39-41]^. Furthermore, the integrity of the brain endothelium is vital for normal self-renewal, proliferation, and differentiation of NPCs^[42]^. In this study, we investigated the role of brain endothelium-derived EV biology in the context of Aβ_40_ pathology to understand the mechanistic dysfunction that may induce aberrant neurogenesis in NPCs.

The level of Aβ_40_ in the CNS is dependent on the balance achieved between endogenous production in the brain, influx from the plasma, and efflux via BBB efflux transporters^[43]^. The relative contributions of these sources to amyloid levels in the brain are difficult to estimate, but they all appear to be critically important. Neurotoxic Aβ_40_ is generated by proteolytic cleavage of the ubiquitously expressed amyloid precursor protein (APP) commonly expressed by neurons^[44]^. Amyloidogenic Aβ peptides range from 30 to 42 amino acids in length, with Aβ_40_ and Aβ_42_ being the two main toxic Aβ species. Aβ_40_ is the main amyloidogenic species, accounting for 90% of all amyloid protein produced. The doses of Aβ_40_ used in this study have pathological relevance as the cerebrospinal fluid Aβ_40_ levels in AD brains are in the nanomolar range^[28]^. 

The first goal of the present study was to evaluate whether differentiation of NPCs could be altered after exposure to EVs containing Aβ_40_. We demonstrated that EV-Aβ_40_ had the ability to alter neurite outgrowth and branching in developing NPCs, prompting us to hypothesize that mitochondrial metabolism was being affected. We also observed a similar decrease in neurite length in a human-induced stem cell-derived neuronal model^[45]^. Generally, mitochondria have a tightly regulated distribution within the cytoplasm of cells; however, when NPCs are rapidly differentiating and require high energetic turnover, the mitochondria are more commonly found in specific growth cones and extensions of these cells. This has broad implications in aiding ATP and calcium release, presynaptic terminal formation, and overall growth of developing neurite extensions. Therefore, we decided to evaluate the impact of EV-associated Aβ_40_ on the mitochondrial morphology and function of NPCs. While we quantitatively confirmed a decrease in the number and density of cristae, we also noticed structural changes to the integrity of individual mitochondria, with areas showing a lack of crista structure or “voids.” These areas with voids may directly correspond to losses in the number, density, and even score of cristae. This observation is significant because it has recently been demonstrated in many pathological studies revolving around organs with a high-energy demand, such as the brain and heart^[46-49]^. This has implications for cell function because it directly corresponds to decreases in mitochondrial membrane permeability, electron transport chain function, and ATP synthesis. 

Next, we used mitochondrial network analysis as an alternative method to assess mitochondrial morphology. Interestingly, the mitochondrial footprint was decreased in EV-Aβ_40_-treated NPCs, while branch length of the networks increased, suggesting a tendency towards a hyperfused state with elongated branches. The presence of a hyperfused state in NPCs is controversial; however, our data suggest that the restricted ability of the organelle to properly divide supports the theory that diseased NPCs have abnormal mitochondria and may exhibit lower ATP production, affecting their potential for self-renewal or differentiation^[50]^.

The mitochondrial membrane is largely impermeable, and the transfer of molecules across it is mediated by channels, pumps, and transporters. Proper assembly of the inner mitochondrial membrane is crucial for maintaining the electron transport chain and ATP bioenergetic production. While NPCs normally maintain glycolysis at low levels, environmental stressors may cause a switch in cell machinery when metabolic respiration is compromised. Indeed, treatment of NPCs with EV-Aβ_40_ and Aβ_40_ alone resulted in a decrease in metabolic respiration. This led us to hypothesize that the metabolism of NPCs is affected by EVs containing Aβ_40_. With a decrease in metabolic respiration and the inability of Aβ_40_-exposed NPCs to switch to compensatory glycolysis, as measured by ECAR levels, we confirmed that overall metabolic regulation in the mitochondria was significantly hindered by EV-Aβ_40_.

The circularity index of a mitochondrion is a relative measure of its shape. Typically, more spherical mitochondria are associated with swelling, while elongated ones result from the dynamic fission and fusion of mitochondria that occur in healthy cells. In our study, NPCs treated with EV-Aβ_40_ showed a tendency for greater fluctuation between spherical and elongated morphology, as well as a decreased respiratory rate. Mitochondrial maintenance is regulated by a balance between fusion and fission^[51,52]^ and a shift in this balance may lead the mitochondria to induce mitophagy or cellular apoptosis^[53]^. The dysregulation of this process can have pathological consequences. This may be particularly important in cells that have high energy demands, such as NPCs, and may have driven the deficits in neurogenesis observed when these cells were exposed to Aβ_40_^[54]^. In general, mitochondrial fusion acts to ameliorate stressful conditions in cells by substituting damaged mitochondria with healthy ones. Meanwhile, mitochondrial fission is necessary to promote mitochondrial division and growth, but can also be used by the cell as a quality control mechanism under pathological conditions to remove damaged mitochondria^[55]^. Fission in the mitochondria is mainly mediated by the GTPase Drp1, which binds to its receptor Fis1 near the endoplasmic reticulum^[56]^. In contrast, mitochondrial fusion is mediated by mitofusin proteins, while the integrity of mitochondrial cristae is maintained by OPA1^[57-59]^. Our data suggest that mitochondrial fusion machinery is dysregulated in NPCs upon exposure to EV-Aβ_40_ and/or Aβ_40_ alone. Indeed, these treatments decreased the expression of OPA1 and Mfn1, while Mfn2 expression was significantly increased. In contrast, the elements of the fission machinery Drp1 and its receptor, Fis1, were significantly downregulated upon treatment with EV-Aβ_40_, supporting our observation of a hyperfused state. Nevertheless, this could also be a compensatory mechanism achieved by cells due to the loss of the integrity of cristae observed in the TEM and metabolic analyses.

Given the irregular mitochondrial dynamics observed in the Aβ_40_-treated cells, we assessed the NPCs for potential engagement in the mitochondrial apoptotic pathway with a focus on Bcl-2 levels. The loss in OPA1 protein expression [Figure 6A] suggests a loss in inner mitochondrial membrane (IMM) fusion and crista structure [Figure 3]. OPA1 is a critical protein for IMM fusion and for preserving crista structure and sequestering cytochrome c in the intermembrane space. A loss of this protein may lead to release of cytochrome c and reduced density of mitochondrial cristae, resulting in white patches or voids in TEM^[60]^. Our results also reveal that a significant decrease in OPA1 levels was associated with a decreased level of Bcl-2, a mitochondrially specific pro-apoptotic inhibitor [Figure 6I-K], an event that can stimulate the release of cytochrome c, which further triggers apoptotic cascades^[61]^.

SIRT3 is a mitochondrially specific NAD-dependent deacetylase that is an essential part of mitochondrial maintenance. Targets of SIRT3 include the electron transport chain, enzymes of intermediary metabolism, and antioxidant defense^[62-64]^. Although functions of SIRT3 in peripheral metabolic regulation have been studied, its action in the brain is not fully understood. One important function of SIRT3 is the deacetylation of NFκB. Thus, decreased activity of SIRT3, as seen in Figure 7A-C, may cause activation of NFκB (acetylated NFκB is active) and induction of downstream inflammatory reactions. In addition, SIRT3 expression is transcriptionally regulated by NFκB^[65]^. Indeed, our data indicate that a decrease in SIRT3 protein expression was associated with the translocation of NFκB into the nucleus in the groups with EV-Aβ_40_ and Aβ_40_ alone. This has implications for future studies on inflammatory and signaling pathways that mediate aberrant neurogenesis.

In summary, the results of the present study indicate that EVs derived from brain endothelial cells can effectively transfer Aβ_40_ to NPCs and affect their neurogenesis via alterations of mitochondrial functions. These events have the potential to modulate the dynamics and severity of AD. Therefore, a better understanding of the interactions between Aβ_40_ and the BBB, and the underlying mechanisms that mediate amyloid accumulation in the brain parenchyma may provide targets for pharmacological intervention. Such interventions may facilitate Aβ removal or protect against entry across the BBB and the development of neurological, neurodevelopmental, and neurocognitive alterations.

## DECLARATIONS

### Availability of data and materials

Available upon request.

### Copyright

© The Author(s) 2022.
