# Supplementary material for: Brain endothelium-derived extracellular vesicles containing amyloid-beta induce mitochondrial alterations in neural progenitor cells
Source: Extracell Vesicles Circ Nucl Acids. 2022 Dec 1;3(4):375–9. doi: 10.20517/evcna.2022.22 (PMC9838065; doi:10.20517/evcna.2022.22)
Supplement: Supplementary file 1 [file evcna-3-4-357-SupplementaryMaterials.pdf]

Osborne *et al. Extracell Vesicles Circ Nucleic Acids* 2022;3:340-62
DOI: 10.20517/evcna.2022.22

Extracellular Vesicles and Circulating Nucleic Acids

Supplementary Material: Brain endothelium-derived extracellular vesicles containing amyloid-beta induce mitochondrial alterations in neural progenitor cells

Supplementary Table 1. Preparation of glutamatergic differentiation D/NPEN media used for iPSC cultures

Reagent	Dilution	Manufacturer	Catalog
Neurobasal media			
DMEM/F12 + L-glutamine			
0.5% N2 supplement	200×		
1% B27 Supplement	100×		
0.5% Non-essential amino acids	200×		
0.5% GlutaMax	200×	Life technologies	35050-061
1% SATO Mix	100×		
1% Insulin-Transferrin-Selenium-A	100×	Life technologies	51300-044
1% Pen/Strep	100×	Life technologies	15140-122
5 ug/mL NAC (N-acetyl cysteine)	1000×	Millipore sigma	A8199
Heparin	2 ug/mL added fresh		

© The Author(s) 2022. Open Access This article is licensed under a Creative Commons Attribution 4.0 International License (https://creativecommons.org/licenses/by/4.0/), which permits unrestricted use, sharing, adaptation, distribution and reproduction in any medium or format, for any purpose, even commercially, as long as you give appropriate credit to the original author(s) and the source, provide a link to the Creative Commons license, and indicate if changes were made.

Supplementary Table 2. List of primary and secondary antibodies used in western blot experiments

Antibody	Dilution	Manufacturer	Species	Catalog
Aβ_{40}	1:1000	Cell signaling	Rabbit	12990
CD63	1:1000	SBI	Rabbit	Exoab-CD63A-1
SIRT3	1:1000	Abcam	Rabbit	Ab217319
Mitofusin1	1:1000	Abcam	Rabbit	Ab129154
Mitofusin2	1:1000	Cell signaling	Rabbit	11925
OPA1	1:1000	Cell signaling	Rabbit	67589
Drp-1	1:1000	Abcam	Rabbit	Ab184247
Bcl-2	1:1000	Cell signaling	Mouse	15071
Anti-GAPDH	1:10,000	Novus biologicals	Mouse	NB600-502FR, NB600-5021
Anti-Mouse 800CW	1:10,000	LI-COR	Donkey	926-32212
Anti-Rabbit 680RD	1:10,000	LI-COR	Goat	926-68071

Supplementary Table 3. List of primary and secondary antibodies used in immunocytochemistry experiments

Antibody	Dilution	Manufacturer	Species	Catalog
Aβ_{40}	1:200	Cell signaling	Rabbit	12,990
Tom20	1:300	Cell signaling	Rabbit	42,406
p65 (NFkB)	1:300	Cell signaling	Rabbit	8242
β-III tubulin	1:500	Cell signaling	Mouse	4466
NeuN	1:500	Abcam	Rabbit	Ab177487
anti-Rabbit IgG (Alexa Fluor 594)	1:500	Thermo Fisher	Goat	A11037

 Osborne *et al. Extracell Vesicles Circ Nucleic Acids* 2022;3:340-62 https://dx.doi.org/10.20517/evcna.2022.22

anti-Mouse IgG (Alexa Fluor 488)	1:500	Thermo Fisher	Goat	A11029
anti-Mouse IgG (Alexa Fluor 647)	1:500	Thermo Fisher	Goat	A32728

`

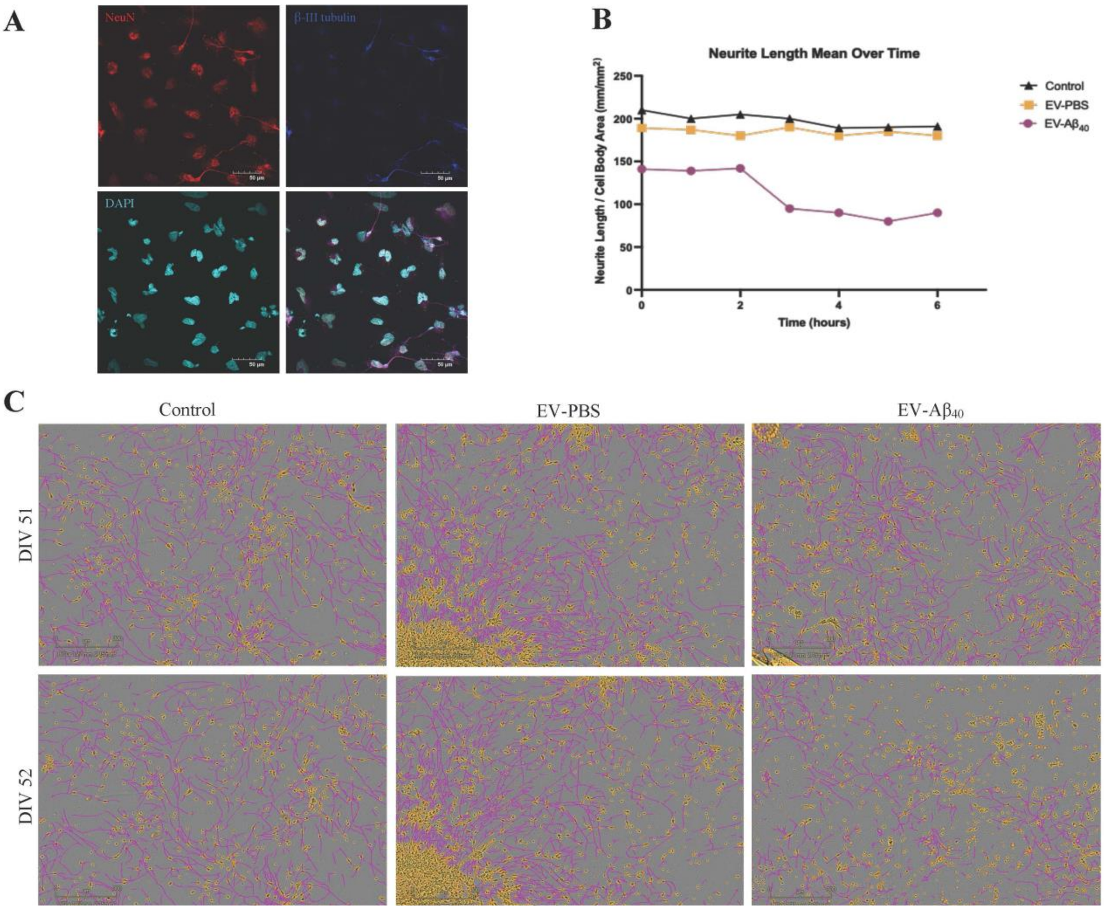

Supplementary Figure 1. iPSC-derived neurons were analyzed for neurite development after treatment with EVs. (A) Human iPSC-derived neurons stained with NeuN for mature neurons, β-III tubulin as a marker for neuronal lineage, and DAPI. Scale bar is 50 μM. Human iPSC-derived glutamatergic neurons were exposed to control EVs or EV-Aβ_{40} for 24 h and imaged on DIV51 and 52. (B) Quantification of IncuCyte images demonstrates the change in neurite length to cell-body area ratio. The total loss in neurite length over time during the first hour after treatment with EVs demonstrates a larger decrease in EV-Aβ_{40} treated neurons. (C) All points represent the average of 9 individual pictures taken in grids in a 6-well plate where pink indicates neurite extensions and yellow represents neuronal cell bodies. *n* = 2 wells per group.
